# A Protective Role by Interleukin-17F in Colon Tumorigenesis

**DOI:** 10.1371/journal.pone.0034959

**Published:** 2012-04-11

**Authors:** Zan Tong, Xuexian O. Yang, Huichao Yan, Weihuang Liu, Xiaoyin Niu, Yun Shi, Wenfeng Fang, Bing Xiong, Yu Wan, Chen Dong

**Affiliations:** 1 School of Basic Medical Science, Wuhan University, Wuhan, China; 2 Department of Immunology, Center for Inflammation and Cancer, M. D. Anderson Cancer Center, Houston, Texas, United States of America; 3 Health Sciences Center, University of New Mexico, Albuquerque, New Mexico, United States of America; 4 Department of Oncology, Wuhan University, Zhongnan Hospital, Wuhan, China; National Cancer Center, Japan

## Abstract

Interleukin-17F (IL-17F), produced by Th17 cells and other immune cells, is a member of IL-17 cytokine family with highest homology to IL-17A. IL-17F has been shown to have multiple functions in inflammatory responses. While IL-17A plays important roles in cancer development, the function of IL-17F in tumorigenesis has not yet been elucidated. In the current study, we found that IL-17F is expressed in normal human colonic epithelial cells, but this expression is greatly decreased in colon cancer tissues. To examine the roles of IL-17F in colon cancer, we have used IL-17F over-expressing colon cancer cell lines and IL-17F-deficient mice. Our data showed decreased tumor growth of IL-17F-transfected HCT116 cells comparing to mock transfectants when transplanted in nude mice. Conversely, there were increased colonic tumor numbers and tumor areas in *Il-17f^−/−^* mice than those from wild-type controls after colon cancer induction. These results indicate that IL-17F plays an inhibitory role in colon tumorigenesis *in vivo*. In IL-17F over-expressing tumors, there was no significant change in leukocyte infiltration; instead, we found decreased VEGF levels and CD31^+^ cells. While the VEGF levels were increased in the colon tissues of *Il-17f^−/−^* mice with colon cancer. Together, our findings demonstrate a protective role for IL-17F in colon cancer development, possibly via inhibiting tumor angiogenesis.

## Introduction

Interleukin-17F (IL-17F) is a pro-inflammatory cytokine produced by activated T cells and other types of immune cells [1], [2]. IL-17F together with 5 other cytokines, including IL-17A, constitute the IL-17 cytokine family [3], [4]. The major source for IL-17A is a new subset of T helper cells, TH17 cells, as well as γδT cells, both of which also produce IL-17F [5]. IL-17F and IL-17A share the strongest amino acid homology and their coding genes locate adjacent to each other on the chromosome [6]. IL-17F could form homodimers or heterodimers with IL-17A in both human and mice [7], [8]. Similar to IL-17A, IL-17F induces the production of many pro-inflammatory cytokines (IL-6/GM-CSF) and chemokines (CXCL1/2/5) in different cell types, as well as enhances granulopoiesis and neutrophil recruitment [9].

IL-17F and IL-17A could utilize the same receptor complexes consisting of IL-17RA and IL-17RC, but different binding affinities and differential expression patterns of IL-17RA and IL-17RC may contribute to overlapping but differential functions of IL-17F versus IL-17A *in vivo* [10]. IL-17F and IL-17A have been reported associated with the pathogenesis of multiple inflammatory diseases, such as asthma and arthritis [11]. In intestinal inflammation, both IL-17F and IL-17A showed up-regulated expression [12], [13]. However, *Il-17f^−/−^* mice were resistant to dextran sulfate sodium (DSS)-induced colitis [14], while *Il-17a* deficiency exacerbated this disease [14], [15]. The basis for this difference is unclear but IL-17F was reported to be expressed by non-lymphoid cells that do not express IL-17A, such as mouse colonic epithelial cells [16].

IL-17A has been found in various tumors and appears to have both pro-tumor and anti-tumor roles depending on the type of tumors and also the existence of host lymphoid system [17]. Some studies showed IL-17A supports tumor growth by facilitating angiogenesis of cervical cancer and lung cancer [18], [19]. In contrast, other reports suggested IL-17A promotes T cell-mediated tumor rejection in fibrosarcoma, hematopoietic immunogenic tumor and lung melanoma [20], [21], [22]. In colorectal cancer samples, clinical research showed increased IL-17A expression [23], [24]. IL-17A deficiency caused significantly reduced intestinal tumorigenesis in Apc^Min/+^ mice [25] and IL-17A blocking antibody could inhibit enterotoxigenic bacteroides fragilis (ETBF)-induced colon carcinogenesis [26]. Therefore, IL-17A may play a pathogenic role in colon cancer. Despite this knowledge, the function of IL-17F in tumorigenesis has been poorly studied.

To address the function of IL-17F in colon cancer, we evaluated IL-17F levels in the human colon cancer tissues and investigated the tumorigenesis by using IL-17F-transfected colon cancer cell lines in nude mice and a chemically-induced colon cancer model in *Il-17f^−/−^* mice. Our results show that IL-17F plays an inhibitory role in colon tumorigenesis *in vivo*.

## Results

### Decreased IL-17F expression associated with human colon cancer

**Figure 1:**
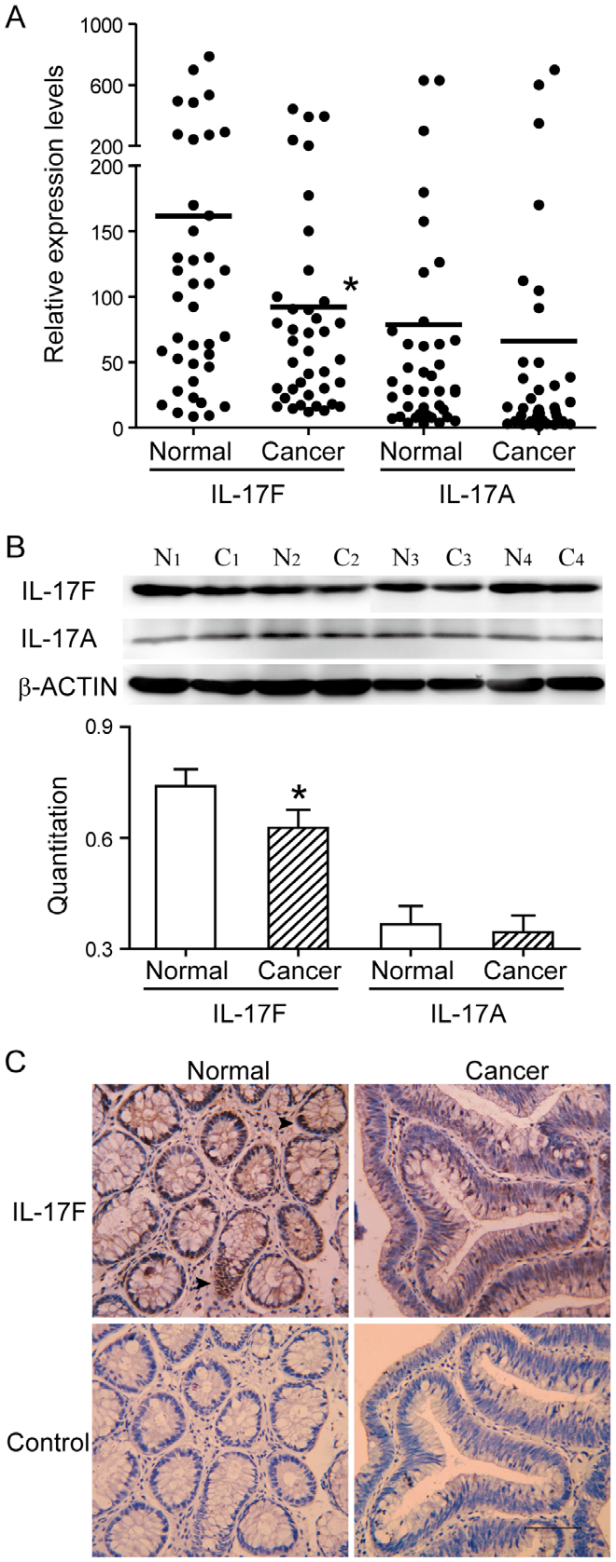
Decreased IL-17F expression associated with human colon cancer. (A) Real-time RT-PCR analysis revealed decreased mRNA levels of *IL-17F* in cancer samples comparing to corresponding normal mucosa, totally 40 pairs of colon samples. (B) Western blot analysis revealed decreased IL-17F expression in cancer samples. Depicted are 4 individual pairs of colon samples. (C) Immunohistochemistry staining of human colon sections using anti-IL-17F antibody. Bar = 50 µm. *, *P*<0.05.

As a first step to determine the function of IL-17F in colon cancer, we analyzed colonic samples from colon cancer patients and found lower mRNA levels of IL-17F in cancer tissues when compared with corresponding normal tissues in the distal margin of the same surgical resection (Figure 1A). Characteristics of the patients are shown (Table S1). Consistent with the mRNA data, we also found lower protein levels of IL-17F in colonic cancer tissues than corresponding normal tissues (Figure 1B). At the same time, we found the mRNA and protein levels of IL-17A showed no significant difference (Figure 1A–B). Interestingly, similar to previously reported expression in mouse colonic epithelial cells, immuno-staining showed that IL-17F was expressed in normal colonic epithelial cells; however, this expression was substantially reduced in malignant epithelial cells (Figure 1C). These data indicated that human IL-17F is expressed in normal colonic epithelial cells but this expression is down-regulated during tumorigenesis, which suggests a role for IL-17F in colonic epithelial tumorigenesis.

### Over-expression of IL-17F inhibits colon tumorigenesis *in vivo*

**Figure 2:**
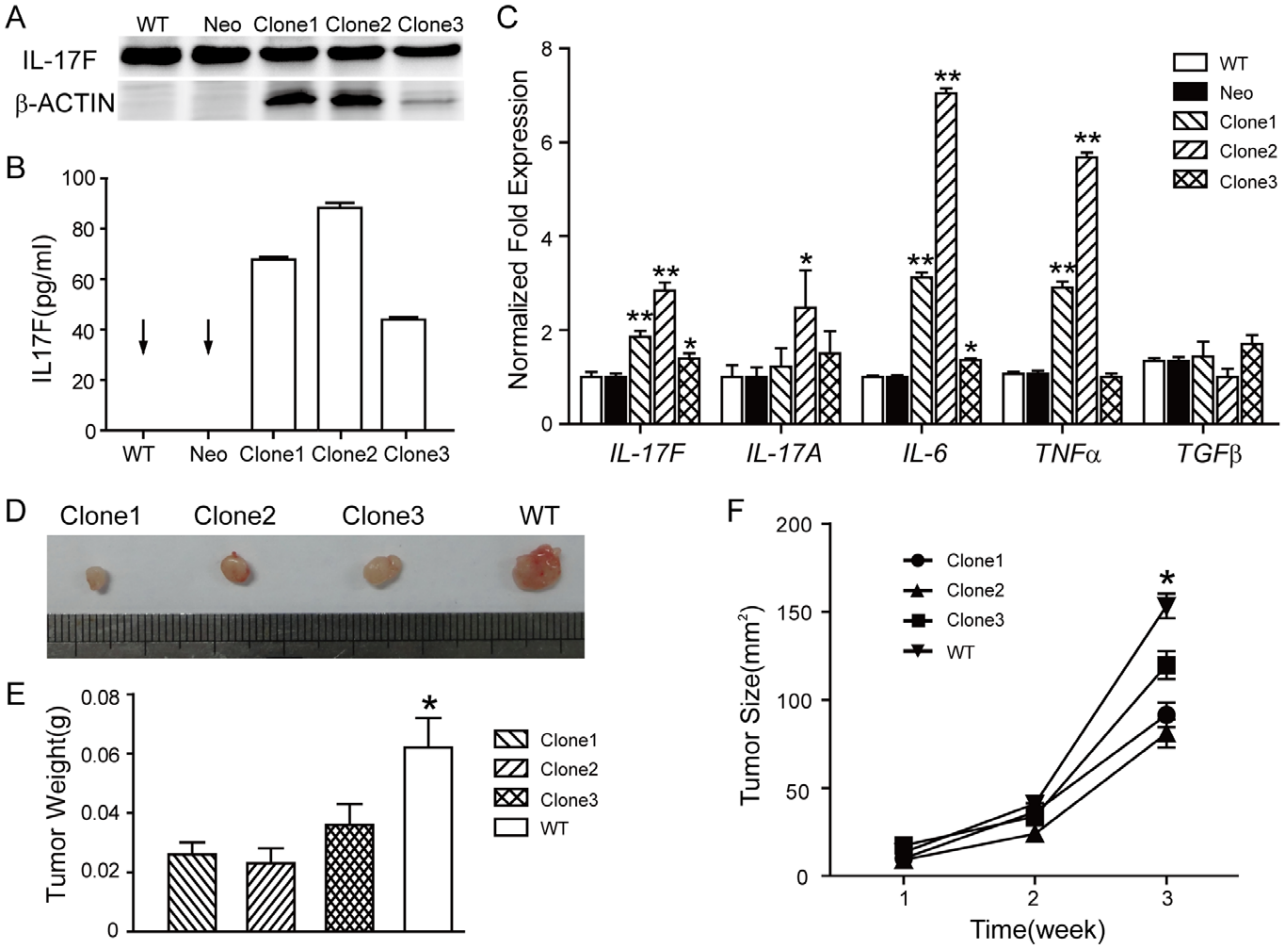
Over-expression of IL-17F inhibits colon tumorigenesis *in vivo*. (A) Western blot analysis of protein lysates from IL-17F-transfected (Clone1, 2, 3), mock-transfected (Neo) and wild-type (WT) HCT116 cells. (B) ELISA analysis of IL-17F in supernatants of the cultured cells. (C) Real-time RT-PCR analysis of the cells. (D) Tumor images and (E) tumor weights on Week 3 after transplantation of IL-17F- or wild-type HCT116 cells into nude mice. (F) Tumor sizes during three weeks after the transplantation (n = 10). *, *P*<0.05. **, *P*<0.001.

To investigate the role of IL-17F in tumorigenesis, we stably transfected colon cancer cell line HCT116 cells with *IL-17F* cDNA and empty vector as control (mock transfectant). Western blot analysis of the cellular proteins (Figure 2A) and ELISA analysis of cultural supernatants (Figure 2B) both showed abundant IL-17F expression in the IL-17F-transfected monoclonal HCT116 cells, while undetectable IL-17F in mock-transfected and wild-type HCT116 cells. Real-time RT-PCR data also showed the mRNA expression of *IL-17F* was remarkably increased in IL-17F-transfected HCT116 cells (Figure 2C). Notably, the mRNA levels of *IL-6* and *TNFα* were also significantly elevated in the IL-17F-transfected group, while those of *IL-17A* were slightly increased (Figure 2C).

We then compared the cell apoptosis, cell cycle and cell growth of the IL-17F-transfected, mock-transfected and wild-type HCT116 cells *in vitro*. FACS analysis showed no significant difference in cell apoptosis and cell cycle among the three groups under normal culture condition (Figure S1A). Slightly increased G0/1 ratio of IL-17F-transfected HCT116 cells after serum starvation for 24 h (Figure S1B). MTT experiments showed identical cell growth (Figure S1C). Soft agar assay showed similar clone formation ability among the three groups (Data not shown). These results revealed that IL-17F may not directly affect apoptosis and proliferation of colon cancer cells *in vitro*.

However, *in vivo*, IL-17F-transfected HCT116 cells grew significantly slower than wild-type cells when transplanted in nude mice (Figure 2D–F). At 3 weeks after transplantation, the mean tumor weights of 0.026 g, 0.023 g, 0.036 g were found in three different IL-17F-transfected tumors, and 0.062 g in wild-type tumors (Figure 2E). During the tumor growth, the mean tumor volumes were increased from 10 at week 1 to 92 mm^3^ at week 3 in IL-17F-transfected clone1 tumors, from 9 to 81 mm^3^ in clone2, and from 17 to 120 mm^3^ in clone3, while 14 to 153 mm^3^ in wild-type tumors during the same period of time (Figure 2F). IL-17F appears to inhibit the tumor growth much more pronouncedly after an initial period in nude mice, suggesting that IL-17F did not affect the initial stage of tumor development but rather suppressed its progression.

### Enhanced colon tumor development in IL-17F-deficient mice

**Figure 3:**
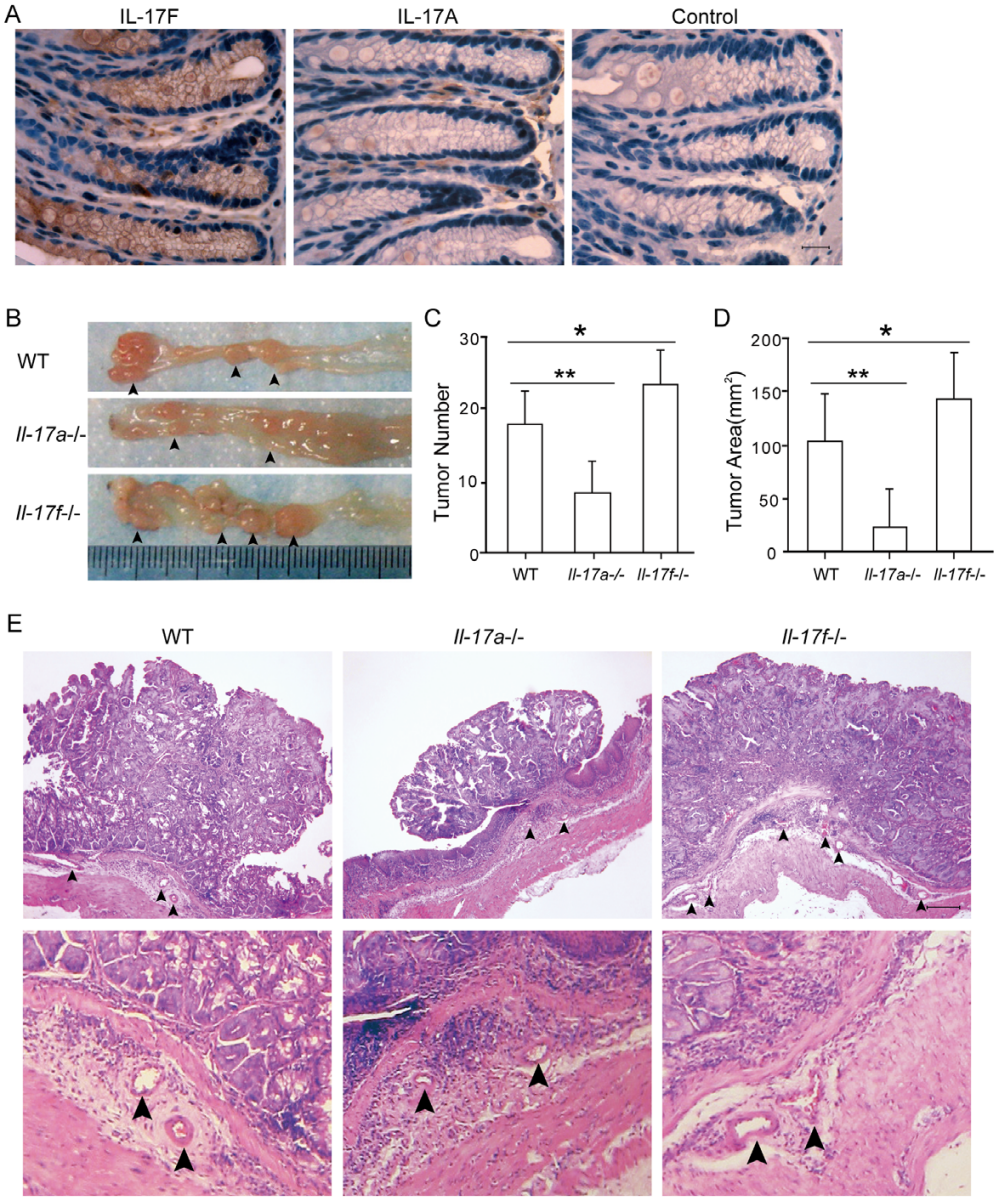
Enhanced colon tumor development in IL-17F-deficient mice. (A) Immunohistochemistry stainings of colon sections from WT mice using anti-IL-17F and IL-17A antibodies. Bar = 10 µm. (B) Tumor images, (C) Tumor numbers and (D) Tumor areas from the colons of AOM-DSS treated WT, *Il-17a^−/−^* and *Il-17f^−/−^* mice. Data shown are a combination of 2 independent experiments (WT, n = 8, *Il-17a^−/−^*, n = 6, and *Il-17f^−/−^*, n = 8). *, *P*<0.05. **, *P*<0.005. (E) H&E stainings of the tumor sections. Arrows pointed out vascular tubes in the basal layer (At least 3 mice from each group were analyzed and representative picture were shown). Bar = 100 µm.

In wild-type mice, IL-17F was mainly produced by colon epithelial cells, while IL-17A was undetectable in colon epithelial cells (Figure 3A). To further evaluate the role of IL-17F in tumorigenesis, we used AOM-DSS induced inflammation-associated colon cancer model in *Il-17f^−/−^*, *Il-17a^−/−^* and wild-type mice. Colons from *Il-17f^−/−^* mice contained significantly more tumor numbers and tumor areas relative to those from wild-type controls (Figure 3B–D), while colons from *Il-17a^−/−^* mice exhibited much fewer tumor polyps and less tumor areas compared with those from wild-type mice. The tumor polyps were sectioned and verified by H&E staining (Figure 3E). The enhanced tumor formation in *Il-17f^−/−^* mice was consistent with inhibited tumor growth by IL-17F over-expression, together revealed an inhibitory role for IL-17F in colon tumorigenesis *in vivo*.

### IL-17F negatively regulates angiogenesis *in vivo*

To find out the possible reason for IL-17F-inhibited tumorigenesis *in vivo*, we analyzed cell frequencies of several immune cell subsets. In HCT116 transplanted tumors, similar cell populations based upon CD11b, Gr-1 and CD49b stainings were observed in IL-17F- and mock-transfected tumors (Figure S2A). In AOM-DSS treated *Il-17f^−/−^*, *Il-17a^−/−^* and WT mice, comparable cell frequencies of IFNγ^+^CD4^+^, IFNγ^+^CD8^+^, and NK1.1^+^ cells in mesenteric lymph nodes (Figure S2B). Collectively, we found no major change in the immune cell subsets by IL-17F, suggesting anti-tumor immunity may not significantly contribute to the reduced colon tumorigenesis in our study.

**Figure 4:**
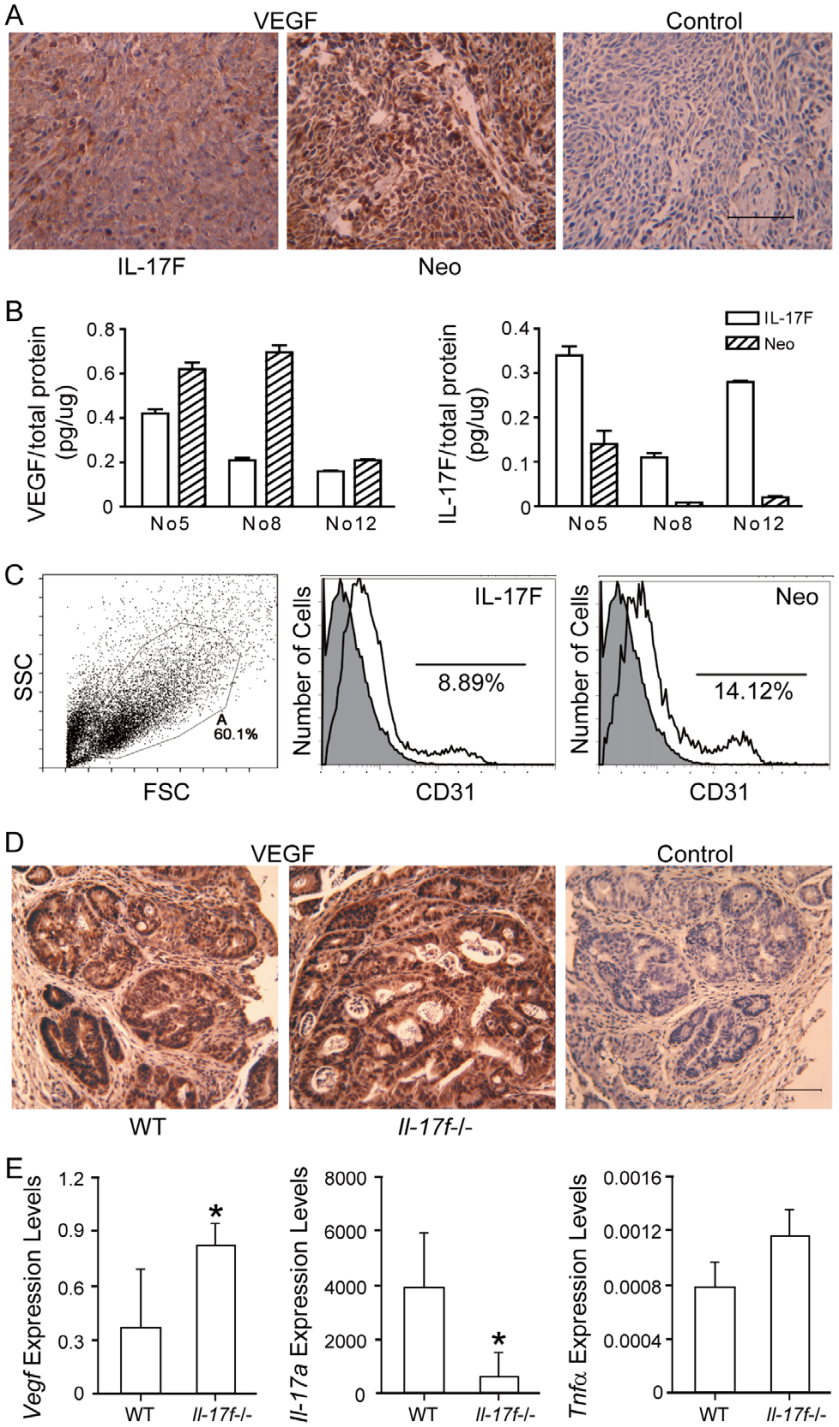
IL-17F inhibits angiogenesis *in vivo*. (A) VEGF staining of transplanted tumors of IL-17F- and mock-transfected HCT116 cells. (B) ELISA analysis of VEGF and IL-17F levels in homogenized tumors from 3 mice (No5, No8, and No12). (C) FACS analysis of CD31 in transplanted tumors after gating on PI negative cells. A representative data was shown (n = 12). (D) VEGF stainings of colon samples from AOM-DSS treated WT and *Il-17f^−/−^* mice. (E) Real-time RT-PCR analysis of the colon samples from AOM-DSS treated WT and *Il-17f^−/−^* mice (n = 4–8). Data shown are a combination of 2 independent experiments. *, *P*<0.05.

The inhibition of IL-17F on the endothelial angiogenesis [1] prompted us to examine the tumor angiogenesis. In the nude mice model, analysis of the tumor sections showed weaker VEGF staining and fewer blood vessels in IL-17F- than mock-transfected HCT116 tumors (Figure 4A). ELISA measurements also showed lower VEGF levels in HCT116-IL-17F tumors (Figure 4B). In FACS analysis of tumors, fewer CD31^+^ cells existed in HCT116-IL-17F (8.9±0.7%) than HCT116-Neo (14.8±1.5%) tumors (Figure 4C). These data suggested inhibited tumor angiogenesis by IL-17F *in vivo*. In the AOM-DSS colon cancer model, there were more vascular tubes in the basal layer of *Il-17f−/−* mice than in that of wild-type mice, while there were very few small vascular tubes in that of *Il-17a−/−* mice (Figure 3E). Analysis of the tumor sections showed stronger VEGF staining in *Il-17f−/−* mice than in wild-type mice (Figure 4D). The mRNA levels of *Vegf* were also increased in the colon tissues of AOM-DSS treated *Il-17f−/−* mice (Figure 4E), while those of *Il-17a* were decreased and *Tnfα* were not significantly changed.

However, we found no difference of *in vitro* VEGF levels (400±21 pg/ml) in the cultural supernatants of the IL-17F-transfected, mock-transfected and wild-type HCT116 cells (Figure S3A). Conditional medium (1/2) from these cells were used to treat HUVEC cells for 24 h, but the cell growth and cell cycle of HUVEC cells showed no change (Figure S3B–C). These results implied that IL-17F may indirectly regulate angiogenesis *in vivo*.

## Discussion

IL-17A has been found with various functions in different tumors and may play a pathogenic role in colon cancer development. However, the function of IL-17F is poorly understood. In this study, by using both over-expression and deletion of IL-17F in colon cancer models, we suggest IL-17F has a protective role in colon tumorigenesis. We found that IL-17F is expressed in normal human colonic epithelial cells, which is down-regulated in colon cancer tissues. Besides, over-expression of IL-17F inhibited the *in vivo* growth of HCT116 cells, an epithelial cell line derived from human colon carcinoma. Therefore, our findings for the first time indicate that IL-17F plays an important role in the tumorigenesis of human colonic epithelial cells.

We found IL-17F inhibited colon tumorigenesis in *vivo*, which is opposite from IL-17A function. Our data showed suppressed colon tumor development of *Il-17a^−/−^* mice in AOM-DSS model, which was consistent with the observations in APC^min/+^ background [25]. It was also reported that over-expression IL-17A in colon cancer cell lines promoted tumor growth in mice [27]. Meanwhile, enhanced tumor formation of *Il-17f^−/−^* mice in AOM-DSS model and reduced tumor growth of IL-17F over-expression cells in nude mice were found in our study. The contrasting effects of IL-17F and IL-17A have also been observed in intestinal inflammation: IL-17F deficiency reduced while IL-17A deficiency exacerbated DSS induced colitis [14], [15]. Considering the complex connections between chronic inflammation and tumorigenesis [28], [29], the two similar but different cytokines possibly form a balance in regulating colonic homeostasis. On the other hand, IL-17a showed increased levels in IL-17F over-expression HCT116 cells and decreased levels in the colon tissues of AOM-DSS treated *il-17f−/−* mice, suggesting a compensatory regulation of IL-17F and IL-17A gene expressions in colon cancer. Further studies are needed, and our study represents a first step toward a better understanding the roles of IL-17F and IL-17A in colonic tumorigenesis.

What's the mechanism underlying the inhibition of colon tumorigenesis by IL-17F? Since IL-17F- and mock-transfected HCT116 cells exhibited similar *in vitro* proliferation kinetics, it is unlikely that IL-17F directly affects *in vivo* proliferation of the transplanted colonic tumors. Our previous work showed Th17 cells promote the activation of tumor specific CD8^+^ T cells [22]. Since IL-17F is an important effecter of Th17 cells, the mechanism involved in our current study is probably a host-depended tumor growth inhibiting effects induced by IL-17F. However, we found no change in leukocyte infiltration in tumors over-expressing IL-17F despite the elevated levels of IL-6 and TNFα *in vitro*, and unaltered cytotoxic immune cell frequencies in mesenteric lymph nodes of colon tumor bearing *Il-17f^−/−^* mice. These results were supported by the report that IL-17F did not affect the migration of mature leukocytes *in vitro* [1].

In the meantime, we found IL-17F-over-expressing tumors had decreased VEGF levels and CD31^+^ cells, which suggested an inhibited angiogenesis. IL-17F suppressed the progression of transplanted tumor but not the initial development. This phenotype is consistent with possible decreased angiogenesis, since angiogenesis is a critical process for the sustained growth of solid tumors. On the other hand, we found more vascular tubes in the basal layer and increased VEGF levels in the colon tumor of *Il-17f−/−* mice. But it's difficult to determine whether the decreased angiogenesis is a cause or effect in primary tumors. Together, our findings suggest that IL-17F suppresses colon cancer development possibly via inhibiting tumor angiogenesis. In support of our results, over-expression of IL-17F in human hepatocellular carcinoma cells was reported with a decrease in tumor size and microvessel density in nude mice [30]. However, we found that IL-17F had no direct effect on the *in vitro* proliferation of vascular endothelial cells (HUVECs) and decreased VEGF production was only found *in vivo* in HCT116 tumor but not *in vitro*. Similarly, IL-17A promoted tumor angiogenesis *in vivo* but failed to exhibit any mitogenic activity for HUVECs *in vitro*; it regulated the production of VEGF depending on cell types *in vitro* [27]. This differential regulation *in vivo and in vitro* is unclear at this point. One possible explanation is that another cell in the tumor microenvironments was regulated by IL-17F/IL-17A in production of VEGF. On the other hand, we found IL-17F dramatically increased TNFα expression in HCT116 cells, and consistent high level of TNFα was reported acted as anti-angiogenic conditions [31]. Together, our data suggest an involvement of IL-17F in the tumor angiogenesis.

In summary, we found that IL-17F is expressed in human colonic epithelial cells and inhibits colon tumorigenesis *in vivo*, possibly by inhibiting angiogenesis. Future study of the mechanism will provide a unique and promising therapeutic approach for colon cancer.

## Materials and Methods

### Clinical sample

Colon cancer tissues and distal normal colonic mucosa were obtained from 40 patients undergoing surgical resection for colon cancer in the Department of Oncology, Zhongnan Hospital of Wuhan University. Cancer tissues and normal tissues were dissected immediately and snap-frozen in liquid nitrogen. All patients signed informed consents for scientific analysis and this study was approved by the Ethical Committee of Wuhan University.

### Animal studies

4–6 week old female BALB/c nude mice were used for transplantation experiments with cancer cells. *Il-17f^−/−^* [14], *Il-17a^−/−^* and wild-type C57BL/6 mice were used for inducing colon cancer by AOM-DSS. All mice were housed in pathogen-free facilities on a 12-h light-dark cycle. All protocols and procedures in this study were approved by the Ethics Committee of Wuhan University (Permit Number: 2010-10007) and M.D. Anderson Cancer Center (Permit Number: 05-05-04233).

### Cell Culture

The HCT116 human colorectal cancer cell line and human umbilical vein endothelial cells (HUVEC) were obtained from the American Type Culture Collection and cultured in DMEM (GIBCO) supplemented with 10%FCS. Human *IL-17F* cDNA was kindly offered by Melissa A.Starovasnik, Genentech.Inc [32] and subcloned into pcDNA3.1vector. HCT116 cells were electroporated with BglII linearized *IL-17F* cDNA construct or pcDNA3.1 vector using Bio-Rad Gene Pulser at 250 V 960 uF. At 48 h after electroporation, transfectants were selected in culture medium supplemented with 800 ug/ml G418 (Merck). G418-resistant monoclones were picked and expanded in the selection medium.

### Tumor growth in nude mice

10^6^ cells were suspended in 200 ul PBS and injected subcutaneously into the lower back of nude mice. IL-17F-transfected HCT116 cells were injected into the right side with vector-transfected HCT116 cells into the left side. Tumor volumes were measured in cubic millimeters with vernier caliper and calculated by the formula tumor size = ab^2^/2. The a is the larger and b is the smaller of the two dimensions. At 3 weeks after transplantation, tumors were separated into two equal parts: one part was used for FACS analysis, the other fixed in 4% paraformaldehyde or snap-frozen in liquid nitrogen.

### AOM-DSS induced colon cancer

*Il-17f^−/−^*, *Il-17a^−/−^* and WT mice were injected intraperitoneally with a initial dose (10 mg/kg AOM in sterile distilled water) of genotoxic colon carcinogen AOM followed by 3 cycles of orally fed DSS (2% in drink water) for 1 week and regular drinking water for 2 weeks as described before [33]. After 9 weeks, colons were collected from the resulting mice and fixed in 4% paraformaldehyde. Numbers and areas of tumor polyps in the colons were analyzed. Mesenteric lymph nodes were collected for FACS analysis.

### Histochemistry

Tumor biopsies were fixed in 4% paraformaldehyde. 5 µm thick paraffin sections were incubated with primary antibodies. Mouse anti-human IL-17F was obtained from R&D, mouse anti-human IL-17A was from ebioscience and rabbit anti-human VEGF was from Santa Cruz Biotechnology. Sections incubated with isotype-matched antibodies were used as negative control. The secondary antibodies were HRP conjugated goat anti-mouse or rabbit IgG. Nuclei were detected by hematoxylin counter-staining.

### FACS analysis of tissue cells

Fresh transplanted tumor biopsies were scissored into small pieces, and separated into single cells using collagenase IV and cell dissociation solution (Sigma). Mesenteric lymph nodes were minced and cells were washed using PBS. Single cells were collected and stained. Different fluorescence conjugated anti-mouse CD11b, Gr-1, CD49b, CD31, CD4, CD8, IFNγ, NK1.1 were from ebioscience.

### ELISA

Cells with the same beginning numbers were cultured in the same volume of medium and the supernatant were collected when cells reached 90% confluence. Tumor tissues were homogenized in PBS and the supernatant were collected after centrifugation. Human IL-17F or VEGF concentrations in the supernatants were determined using Elisa kits as recommended by the assay manufacturer. At the same time, protein concentrations in the supernatants were determined by BCA protein assay kit (pierce).

### Quantitative RT-PCR

RNA were extracted using Trizol method and reverse transcripted to cDNA using RT Kit (Invitrogen). Quantitative PCR analyses were performed on Bio-Rad CFX96 System using the SYBR Green PCR Master Mix (Takara). The relative RNA level of each gene was normalized against *β-ACTIN*. Primers used for human genes: *IL-17F*: 5′-ACC CCT CGG AAG TTG TAC-3′ and 5′-CAG TCA CCA GCA CCT TC-3′. *IL-17A*: 5′- GAA GTT CTG GGA GGA GAC ATT G-3′ and 5′- GGA GTG TTG CTT GAG GAA GAG-3′. *IL-6*: 5′- ACA ACT CAT CTC ATT CTG C-3′ and 5′- GTG TCC TAA CGC TCA TAC-3′. *TNFα*: 5′- GGT ATG AGC CCA TCT ATC TG-3′ and 5′- GCA ATG ATC CCA AAG TAG AC-3′. *TGFβ*: 5′-CCC ACA ACG AAA TCT ATG AC-3′ and 5′-TGA GGT ATC GCC AGG AAT-3′. *β-ACTIN*: 5′-GCG TGA CAT TAA GGA GAA G-3′ and 5′-GAA GGA AGG CTG GAA GAG-3′. Primers used for mouse genes: *Il-17a:* 5′-CTC CAG AAG GCC CTC AGA CTA C-3′ and 5′-GGG TCT TCA TTG CGG TGG-3′. *Tnfa:* 5′-GAC CCT CAC ACT CAG ATC ATC-3′ and 5′-CGC TGG CTC AGC CAC TC-3′. *Vegf:* 5′-TCC CAG GCT GCA CCC ACG ACA G-3′ and 5′-TGA CGT GGG CAC GCA CTC CAG G-3′. β-actin: 5′-TGA AGA TCA AGA TCA TTG CTC CTC-3′ and 5′-CCT GCT TGC TGA TCC ACA TC-3′.

### Western blot analysis

40–60 µg protein were loaded on SDS-12% PAGE Gel. Blots were probed with mouse anti-human IL-17F or IL-17A, and anti-human β-ACTIN as loading control. Signal intensities in the blots were measured by Quantity One software and transformed to the bar graphs.

### Statistical analysis

All values are expressed as mean ± standard error of the mean. Differences were analyzed using student's *t*-test and *p*< = 0.05 considered statically significant.

## Supporting Information

Figure S1 **IL-17F does not affect cell apoptosis, cell cycle and cell growth** ***in vitro*** **.** FACS analysis of IL-17F-transfected, mock-transfected and wild-type HCT116 cells cultured in 10%FBS medium (A) or 0.1%FBS medium (B). (C) MTT analysis of IL-17F-transfected, mock-transfected and wild-type HCT116 cells.
(TIF)

Figure S2 **IL-17F does not change immune cell subsets** ***in vivo*** **.** (A) FACS analysis of CD11b, Gr-1, and CD49b in transplanted tumors of IL-17F- and mock-transfected HCT116 cells. (B) IFNγ^+^CD4^+^, IFNγ^+^CD8^+^ and NK1.1^+^ cell frequencies in mesenteric lymph node cells from AOM-DSS treated WT, *Il-17a^−/−^* and *Il-17f^−/−^* mice.
(TIF)

Figure S3 **IL-17F does not influence cell growth and cell cycle of HUVEC cells** ***in vitro*** **.** (A) Elisa analysis of VEGF levels in supernatants of IL-17F-transfected, mock-transfected and wild-type HCT116 cells. MTT analysis (B) and FACS analysis (C) of HUVEC cells cultured in conditioned medium (from IL-17F-transfected, mock-transfected or wild-type HCT116 cells) or normal medium.
(TIF)

Table S1 **Clinical and pathological characteristics of patients with colorectal cancer.**
(DOC)
