# Supplementary figures and images for: A Protective Role by Interleukin-17F in Colon Tumorigenesis
Source: PLoS One. 2012 Apr 11;7(4):e34959. doi: 10.1371/journal.pone.0034959 (PMC3324558; doi:10.1371/journal.pone.0034959)

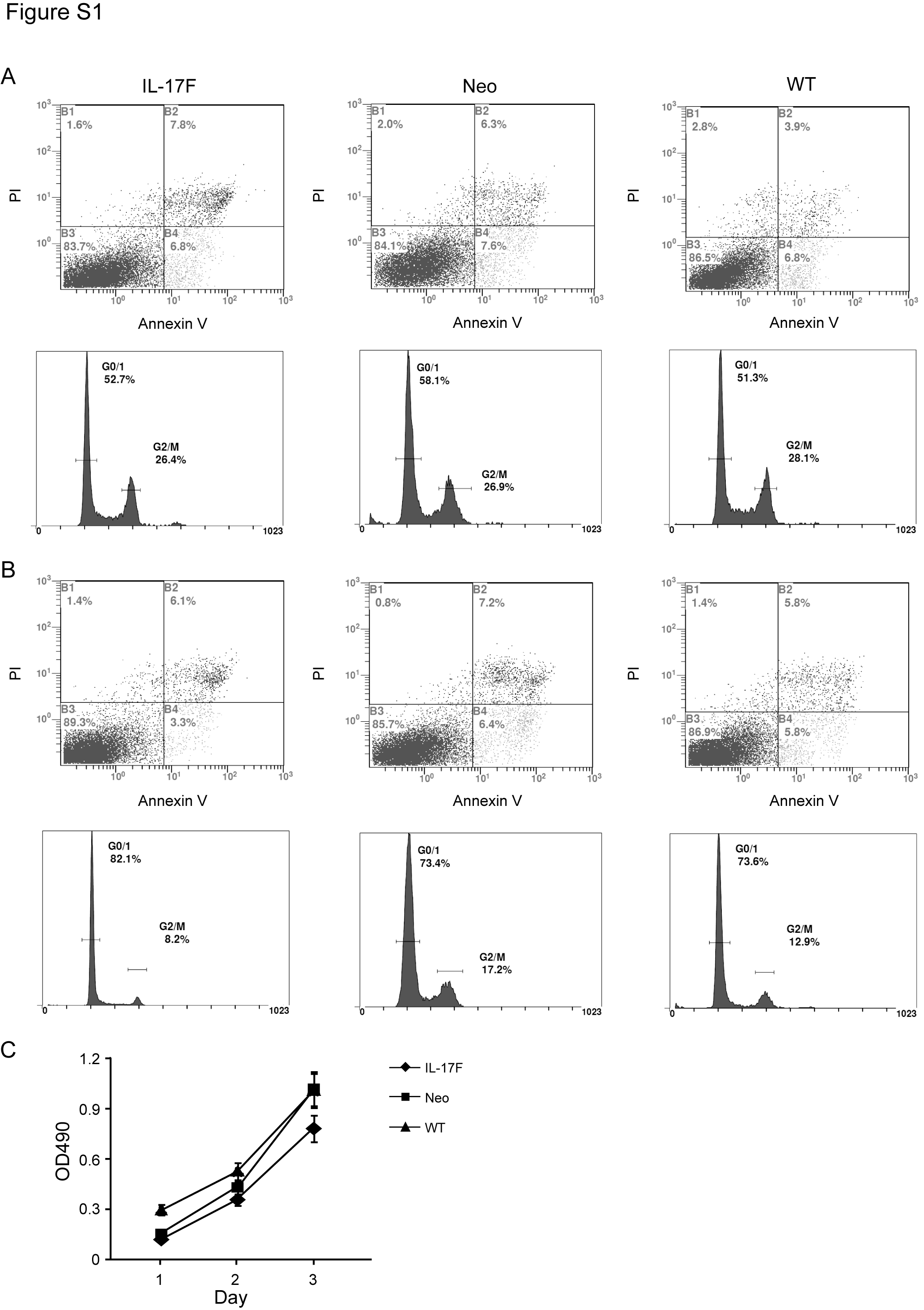

Supplement: Figure S1 — IL-17F does not affect cell apoptosis, cell cycle and cell growth in vitro . FACS analysis of IL-17F-transfected, mock-transfected and wild-type HCT116 cells cultured in 10%FBS medium (A) or 0.1%FBS medium (B). (C) MTT analysis of IL-17F-transfected, mock-transfected and wild-type HCT116 cells. (TIF) [file pone.0034959.s001.tif]

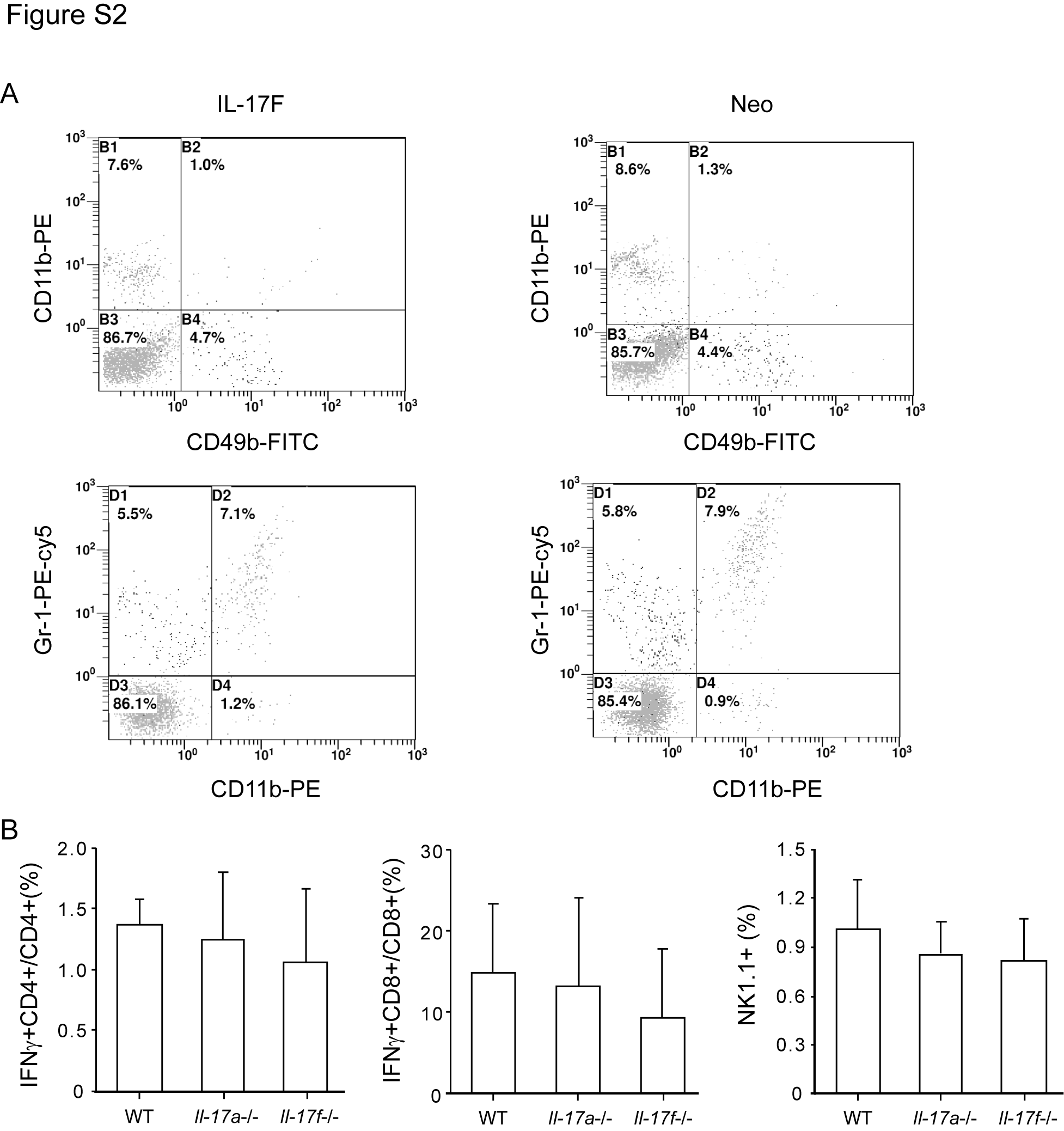

Supplement: Figure S2 — IL-17F does not change immune cell subsets in vivo . (A) FACS analysis of CD11b, Gr-1, and CD49b in transplanted tumors of IL-17F- and mock-transfected HCT116 cells. (B) IFNγ+CD4+, IFNγ+CD8+ and NK1.1+ cell frequencies in mesenteric lymph node cells from AOM-DSS treated WT, Il-17a−/− and Il-17f−/− mice. (TIF) [file pone.0034959.s002.tif]

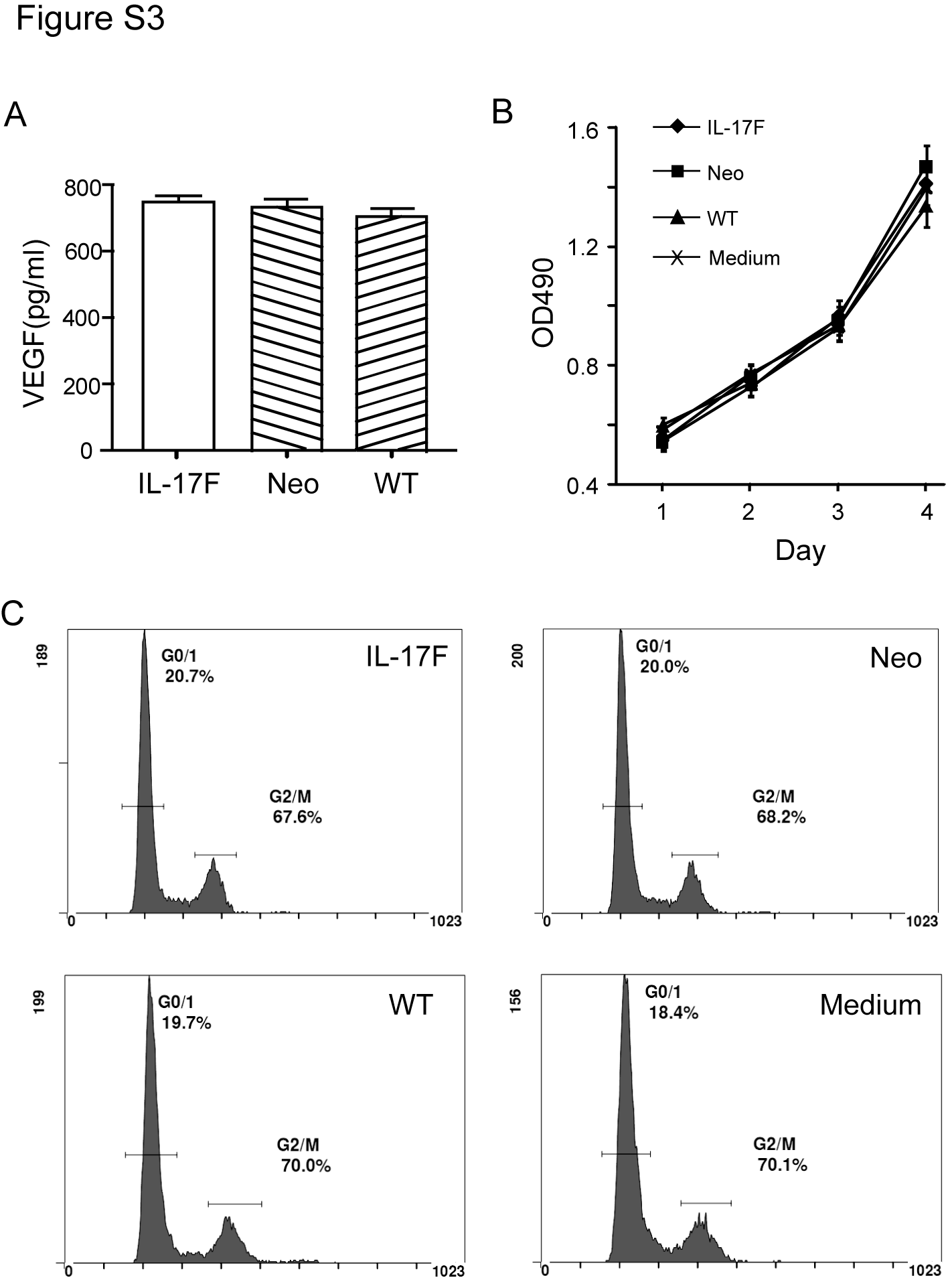

Supplement: Figure S3 — IL-17F does not influence cell growth and cell cycle of HUVEC cells in vitro . (A) Elisa analysis of VEGF levels in supernatants of IL-17F-transfected, mock-transfected and wild-type HCT116 cells. MTT analysis (B) and FACS analysis (C) of HUVEC cells cultured in conditioned medium (from IL-17F-transfected, mock-transfected or wild-type HCT116 cells) or normal medium. (TIF) [file pone.0034959.s003.tif]
