# Supplementary material for: A Protective Role by Interleukin-17F in Colon Tumorigenesis
Source: PLoS One. 2012 Apr 11;7(4):e34959. doi: 10.1371/journal.pone.0034959 (PMC3324558; doi:10.1371/journal.pone.0034959)
Supplement: Table S1 — Clinical and pathological characteristics of patients with colorectal cancer. (DOC) [file pone.0034959.s004.doc]

Table S1. Clinical and pathological characteristics of patients with colorectal cancer.

| Characteristics | Subcategory | Number |
| --- | --- | --- |
| Sex | Female | 18 |
|  | Male | 22 |
| Age(y) | >55 | 24 |
|  | <=55 | 16 |
| Location | Proximal colon | 22 |
|  | Distal Colon+Rectum | 18 |
| Dukes' stage | A | 9 |
|  | B | 17 |
|  | C | 10 |
|  | D | 4 |
| Lymph node involvement | Yes | 14 |
|  | No | 26 |
